# Efficacy and safety of fecal microbiota transplantation *via* colonoscopy as add-on therapy in patients with mild-to-moderate ulcerative colitis: A randomized clinical trial

**DOI:** 10.3389/fmed.2022.1049849

**Published:** 2023-01-12

**Authors:** Sergii Tkach, Andrii Dorofeyev, Iurii Kuzenko, Tetyana Falalyeyeva, Olena Tsyryuk, Oleksandr Kovalchuk, Nazarii Kobyliak, Ludovico Abenavoli, Luigi Boccuto

**Affiliations:** ^1^Ukrainian Research and Practical Centre of Endocrine Surgery, Transplantation of Endocrine Organs and Tissues of the Ministry of Health of Ukraine, Kyiv, Ukraine; ^2^Shupyk National Medical Academy of Postgraduate Education, Kyiv, Ukraine; ^3^Medical Laboratory CSD, Kyiv, Ukraine; ^4^Educational-Scientific Center, Institute of Biology and Medicine, Taras Shevchenko National University of Kyiv, Kyiv, Ukraine; ^5^Endocrinology Department, Bogomolets National Medical University, Kyiv, Ukraine; ^6^Department of Health Sciences, University “Magna Graecia”, Catanzaro, Italy; ^7^Healthcare Genetics Program, School of Nursing, Clemson University, Clemson, SC, United States; ^8^Clemson University School of Health Research, Clemson, SC, United States

**Keywords:** fecal microbiota transplantation, gut microbiota, dysbiosis, ulcerative colitis, inflammatory bowel disease

## Abstract

**Introduction:**

Growing evidence supports the effectiveness of fecal microbiota transplantation (FMT) in treating ulcerative colitis (UC), although its effects seem to depend on the method of introduction, the number of procedures, the donor material, and the severity of UC.

**Aim:**

This study aimed to assess FMT's clinical and microbiological efficacy, tolerability, and safety in patients with mild-to-moderate UC.

**Material and methods:**

Patients with mild-to-moderate UC were randomized into two groups. The first group (standard-care, *n* = 27) was treated with basic therapy–mesalazine–at a daily dose of 3 g (2 g orally + 1 g rectally). In the second group (FMT group, *n* = 26), while taking mesalazine at the indicated dose, each patient with UC as add-on therapy underwent a single FMT procedure with fresh material delivered by colonoscopy from a healthy donor. The clinical efficacy of treatment in both groups was evaluated after 4 and 8 weeks. The primary outcome was remission of UC, defined as a partial Mayo score ≤2, and decreased fecal calprotectin. All patients underwent bacteriological examination of feces for quantitative microbiota composition changes.

**Results:**

Clinical response in the form of a significant decrease in stool frequency and a tendency to normalize its consistency after 4 weeks was detected in 14 (51.9%) patients of the standard care group and 16 patients (61.5%) of the FMT group (*p* = 0.583). The Mayo score in the standard care group was 3.59 ± 1.21 and in the FMT group−3.15±1.04 (*p*=0.166). After 8 weeks, the main primary endpoint was achieved in 70.4% of the standard-care group patients as compared to 84.6% of participants who received FMT as add-on therapy (*p* = 0.215). A more pronounced decrease in Mayo score was observed in the FMT group compared to the standard-care group (1.34 ± 1.44 vs. 2.14 ± 1.4; *p* = 0.045). All patients also showed a significant decrease in fecal calprotectin levels, which correlated with clinical data, stool frequency, and clinical remission. An improvement in gut microbiota composition was noted in both groups, albeit it was significantly more pronounced in the FMT group.

**Conclusions:**

FTM in patients with mild-to-moderate UC is a well-tolerated, effective, and safe method of treatment in comparison to basic therapy.

**Clinical trial registration:**

https://clinicaltrials.gov/ct2/show/NCT05538026?term=kobyliak&draw=2&rank=4, identifier: NCT05538026.

## Introduction

Ulcerative colitis (UC) is a chronic immune-mediated inflammatory bowel disease (IBD) that almost always affects the rectum and often extends to the more proximal colon. UC usually begins at a young age (15–30 years) and most patients (~85%) present with mild or moderate activity, characterized by periods of exacerbation and remission (1).

The severity of UC can be mild, moderate, or severe, with definitions of disease activity varying in clinical practice and the medical literature. Since it is not always possible to clearly distinguish between the severity of the course, in recent years, there has been a tendency to distinguish between mild-to-moderate and moderate-to-severe forms of UC (1). Mild-to-moderate UC is significantly more common, typically characterized by <4–6 bowel movements per day, mild/moderate stool bleeding, no significant symptoms, low overall inflammatory response, and no evidence of high inflammatory activity based on both the Truelove and Witt criteria and the Mayo clinics ones (1–3).

More than 90% of patients with UC after diagnosis begin treatment with 5-aminosalicylic acid (5-ASA), achieve clinical remission in a fairly short time, and then continue to take these medications to maintain remission (4). Fewer patients with more severe diseases require the use of immunomodulators or biological therapy.

The etiology of UC is not exactly known, although it is multifactorial, and both genetic and environmental factors contribute to its development (5). In recent years, special attention in the study of the mechanisms of development of UC has been paid to the study of the gut microbiome (GM) (6, 7). The data available to date suggest that certain changes in GM can induce disturbances in key links in the pathogenesis of UC: local and systemic immune response, the state of the intestinal mucosal barrier, features of its permeability, and changes in the morphological structure (8–11). It is possible that the severity of gut dysbiosis in patients with UC largely determines the clinical picture of the disease, the severity of exacerbation, and the stability of remission (9). Therefore, the assessment of the impact of various types of UC treatment on changes in GM is of particular interest.

Considering the important pathogenetic role of gut dysbiosis, additional strategies for treating UC have recently been focused on the modification of altered GM using various drug and non-drug methods (12). One such method is fecal microbiota transplantation (FMT), consisting of the simultaneous replacement of the GM of a sick recipient with fecal material from a healthy donor (13). Even though so far the only officially approved indication for FMT is recurrent *Clostridium (C) difficile* infection, the effectiveness of FMT is currently being studied in treating other gastrointestinal and non-gastrointestinal disorders (14–17), and several studies have been conducted to specifically study the effectiveness of FMT in UC, showing encouraging results (18–24).

This study aimed to assess FMT's clinical and microbiological efficacy, tolerability, and safety in patients with mild-to-moderate UC.

## Materials and methods

### Study design

This open-label, single-center, randomized clinical study was conducted to examine the effectiveness of FMT as add-on therapy in patients with a confirmed clinical diagnosis of mild-to-moderate UC. The study protocol was designed in compliance with principles of the Declaration of Helsinki 1975. The study was approved by the Ethics Committee at Ukrainian Research and Practical Center of Endocrine Surgery, Transplantation of Endocrine Organs and Tissues of the Ministry of Health of Ukraine (protocol number: 6/2020) and was registered in the ClinicalTrial.gov database under entry number NCT05538026. Before RCT was initiated, its purpose and methods were discussed with participants and all patients voluntarily signed the informed consent.

Depending on the treatment, all patients with mild-to-moderate UC were randomized into 2 groups using a computer random number method in a ratio of 1:1. Randomization was carried out by an expert in statistics with blocks of four using a computer-generated list at www.randomization.com. The groups were homogeneous in terms of age, gender, and diagnosis. The patients in the first group (standard-care, *n* = 27) were prescribed basic therapy, mesalazine (Pentasa), at a daily dose of 3 g (2 g orally + 1 g rectally). In the second group (FMT group, *n* = 26), while taking mesalazine at the indicated dose, each patient with UC as add-on therapy underwent a single FMT procedure with fresh material from a healthy donor.

### Participants selection

Participants were eligible for inclusion in the trial if they had a verified endoscopically and histologically UC. The severity and degree of activity for UC were assessed based on the Mayo score, which is one of the most commonly used disease activity indices in placebo-controlled trials in UC (25). In its complete form, it is composed of four parts: rectal bleeding, stool frequency, physician assessment, and endoscopy appearance. Each part is rated from 0 to 3, giving a total score of 0–12. A partial Mayo score (eliminates endoscopy) of 2–4 points indicates mildly active disease, a score of 5–6 points indicates moderately active disease, and a score of 7–9 points indicates severely active disease (26). Eligible patients were with active mild-to-moderate UC (defined as a partial Mayo score of 4–6, and a Mayo endoscopic subscore ≥1). Other inclusion criteria were as follows: adult patients (age: 18–60 years); negative results of stool culture for the presence of pathogenic bacteria (*Shigella* spp., *Salmonella* spp., *Campylobacter* spp., *Yersinia* spp.) and toxin-producing *C. difficile*; treatment with mesalazine at a daily dose of 3 g during the last 4 weeks; fecal calprotectin over 150 μg/g and a signed informed consent form.

Patients were excluded if pregnant or breastfeeding; with previous surgery on the abdominal cavity; with severe current disease (hepatic, renal, respiratory, or cardiovascular); with corticosteroids, biological agents, probiotic, or antibiotic use within 8 weeks prior to study initiation; or any condition or circumstance that would, in the opinion of the investigator, prevent completion of the study or interfere with the analysis of study results.

### Procedure

For our FMT procedures, we used fecal material from one donor tested in accordance with the European Consensus on FMT that was published in the form of clinical guidelines for physicians in 2017 (19). A healthy 39-year-old Caucasian male was recruited as a donor, since he had no harmful habits, adhered to a healthy lifestyle, and had a BMI of 24.5 kg/m^2^. His fecal material has already been utilized in other FMTs that have proven effective in the treatment of recurrent *C. difficile* infection. The donor underwent a physical examination, as well as studies and blood tests to exclude pathology of the gastrointestinal tract, metabolic or neurological disorders (complete blood count, blood glucose, electrolytes, and inflammatory markers), liver tests, and thyroid function tests, as well as serological screening tests for HIV, syphilis, and viral hepatitis A, B, and C. The results of his stool culture for the presence of pathogenic bacteria (*Shigella* spp., *Salmonella* spp., *Campylobacter* spp., *Yersinia* spp. and toxin-producing *C. difficile*), rotaviruses, helminth eggs, and parasites were also negative. His stool culture indicated the absence of gut dysbiosis. Donor fecal samples were tested every 2 months and remained normobiotic with minor variations in the quantitative composition of gut bacteria.

FMTs were prepared as follows: 50–80 g of freshly delivered feces were mixed with 200 mL of isotonic saline and 50 mL of 85% glycerol, homogenized in a blender for 60 s, filtered through a 0.5 mm mesh steel strainer, drawn on 50 mL sterile Luerlock syringes, and sealed.

An appropriately prepared fresh stool suspension from a healthy donor was administered to all patients a single time during a colonoscopy (through a probe inserted into the working channel of the endoscope) while patients were under the effects of intravenous anesthesia.

### Outcomes assessment

All patients underwent a comprehensive laboratory and instrumental examination, including general clinical and biochemical blood tests (liver function tests, thyroid hormones, serological examination for celiac disease, electrolytes), fecal examination for calprotectin, helminth eggs and parasites, abdominal ultrasonography, gastroduodenoscopy and colonoscopy with segmental biopsy.

Evaluation of the clinical efficacy of treatment in both groups was carried out after 4 weeks and 8 weeks. The primary outcome was remission of UC, defined as a partial Mayo score ≤2, and decreased fecal calprotectin.

All patients underwent bacteriological examination of feces for quantitative microbiota composition changes in terms of secondary outcome. The gut microbiota of all patients was studied before and 1 month after FMT at the level of the main microbial phylotypes by determining the DNA *Firmicutes, Bacteroidetes*, and *Actinobacteria* in stool samples using a quantitative real-time polymerase chain reaction (PCR) (qRT-PCR). For this, samples of fresh feces were placed in a special container by each patient. An aliquot of feces was taken within 10 min after defecation, immediately frozen, and stored at −20°C until DNA isolation using the phenol-chloroform method according to protocol. DNA was eluted in 200 μl of buffer, and the amount and quality of DNA were measured using a NanoDrop ND-8000 (Thermo Scientific, USA). Samples with a DNA concentration of fewer than 20 ng or with a 260:280 fluorescence ratio of <1.8 were either subjected to ethanol precipitation to become concentrated or further purified according to quality standards. Various taxa were quantified by qPCR using primers targeting the 16S rRNA gene specific for *Firmicutes, Actinobacteria, Bacteroidetes*, and *Faecalibacterium (F) prausnitzi*, as well as universal primers. Genotyping was performed *via* qRT-PCR using the primer structure and temperature cycle parameters.

One of the problems of the PCR approach is related to normalization. To address this issue, the set could be extended by adding a universal pair of primers (and a probe) targeting total prokaryotic content that can be used for normalization purposes (for example, by dividing signals from other taxa by it). Although this would reflect the microbial concentration in the analyzed DNA sample, this concentration could not directly correspond to the concentration in the subject's stool, as it can change considerably during the extraction (27).

Adverse reactions due to FMT were assessed daily over a period of 3 days, and then weekly over the trial.

### Sample size calculation

The sample size was calculated using WINPEPI 11.65 (Brixton Health, Israel) software based on the previously published study (21). We calculated that to allow for dropouts at 10% we would need 60 participants in a balanced two-group design (α = 0.05; 1-β = 0.80).

### Statistical analysis

Statistical analysis was performed using the standard software SPSS version 20.0 (SPSS, Inc., Chicago, Illinois) and GraphPad Prism, version 6.0 (GraphPad Software, Inc., La Jolla, CA, USA). Analyses were done according to the intention-to-treat principle, excluding participants without data from the analyses of all clinical endpoints, who did not undergo treatment, and participants diagnosed with any other disease at 8 weeks. Quantitative changes were presented as the mean and standard deviation (M ± SD), and qualitative changes were presented as %. In order to prove the normal distribution hypothesis, Kolmogorov-Smirnov one-sample test was used. To estimate the difference in the incoming quantitative data χ^2^ criterion was used. A paired *t*-test and a repeated measure analysis of variance (RM-ANOVA) were used to determine, within each group, the difference between the initiation of therapy and the 4 weeks and end of the trial. The changes in outcomes of the participants after the initiation of therapy and the end of the trial were compared by paired sample *t*-tests. Analysis of covariance (ANCOVA) was used to identify any differences between the two groups after the intervention, adjusting for baseline values. Differences between groups were considered significant at a value of *p* < 0.05.

## Results

**Figure 1:**
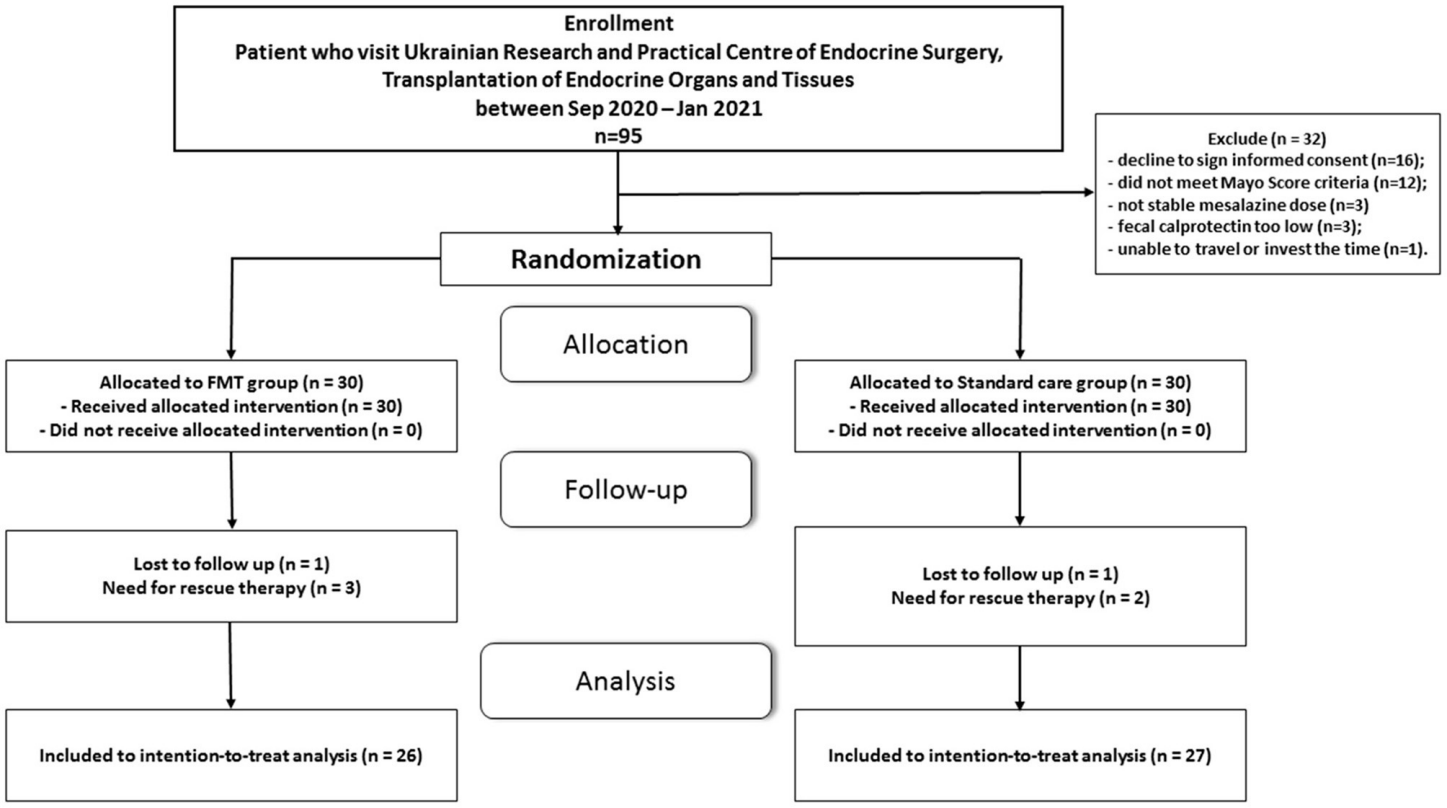
Consolidated standards of reporting trials (CONSORT) flow chart-trial protocol.

Recruitment for a single-center open comparative randomized clinical trial was started in September 2020 and continued until January 2022 at the Ukrainian Research and Practical Center of Endocrine Surgery, Transplantation of Endocrine Organs and Tissues of the Ministry of Health of Ukraine. For the primary analysis, 95 patients were selected. After carefully considering compliance with the inclusion/exclusion criteria, 18 patients were not eligible. The main reasons were low fecal calprotectin (*n* = 3), not stable mesalazine dosage (*n* = 3), and 12 patients who did not meet Mayo score criteria (Figure 1). An in-person consult with all other potential participants allowed us to explain the study criteria, purpose, and methodology of the study. After consideration of the proposal, 16 patients refused to give their informed consent, and 1 was unable to travel or invest the time. At the end of the enrolment period, with possible bias adjustment, 60 patients with mild-to-moderate UC were chosen to be included in the study. All patients were equally distributed in a random order to FMT or standard care group. One randomly assigned participant in both groups withdrew their informed consent without explanation. Moreover, 5 patients (3 in FMT and 2 in standard care group) needed rescue therapy with steroids after initiation of intervention. This left 53 participants for the final modified intention-to-treat analysis. A CONSORT flow chart with a general protocol schedule is shown in Figure 1.

The enrolled patients' baseline demographic and clinical characteristics did not significantly differ between groups (Table 1). A total of 53 patients (32 women, 21 men) with active mild/moderate UC were examined. The severity of UC was assessed based on the Mayo score and fecal calprotectin level. The partial Mayo score at baseline in patients of the standard care group was 5.03±0.8, which does not differ from FMT−5.00±0.8 points (*p* = 0.868) (Table 1). The level of fecal calprotectin in patients with UC before treatment was 265.12 ± 47.63 in standard care and 256.36 ± 47.68 μg/g in the FMT group (*p* = 0.507).

**Table 1:** Baseline clinical parameters in examined patients (M ± SD or %).

**Baseline characteristics**	**Standard care group (*n* = 27)**	**FMT group (*n* = 26)**	***p***
Gender (male/female)	10/17	11/15	0.456
Age, years	40.1 ± 12.1	42.4 ± 11.4	0.360
UC duration, years	5.11 ± 2.39	5.81 ± 2.2	0.276
Smoking status, *n* (%)	9 (33.3%)	11 (42.3%)	0.500
Body mass index (BMI), kg/m^2^	25.67 ± 2.68	25.26 ± 3.19	0.619
Mayo index, points	5.03 ± 0.80	5.00 ± 0.80	0.868
Fecal calprotectin, μg/g	265.12 ± 47.63	256.36 ± 47.68	0.507
Endoscopic severity index, points	6.78 ± 0.75	6.69 ± 0.89	0.903
**Localization**			
Proctitis	2	1	
Proctosigmoiditis	12	14	
Left-sided colitis	13	11	

### Primary outcomes analysis

**Figure 2:**
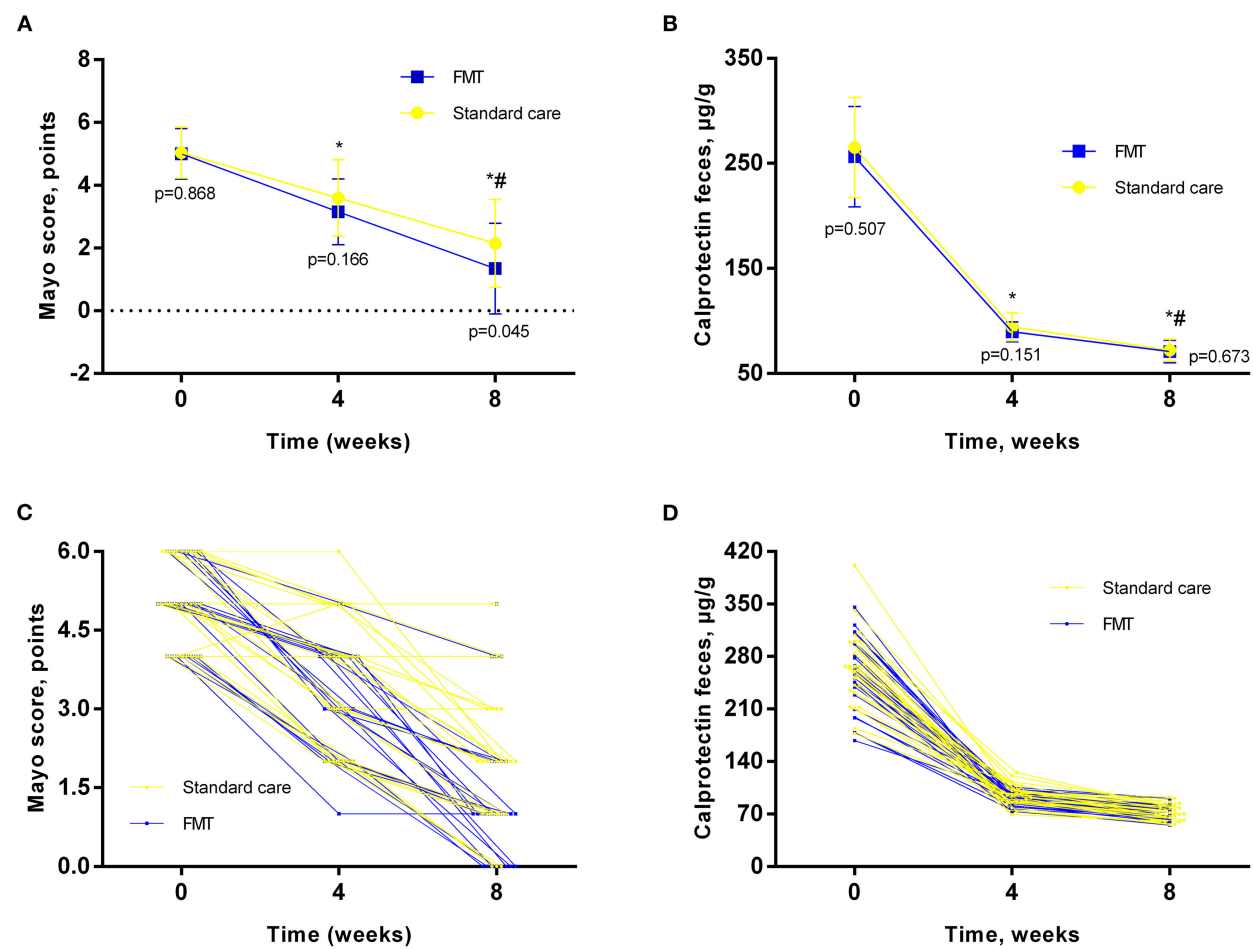
Main outcomes analysis in different timepoints. **(A, C)**–Mayo Score; **(B, D)**–fecal calprotectin; Data expressed as mean ± SD **(A, B)** and individual values **(C, D)**. RM-ANOVA was used to identify any differences within groups. *p* – indicates the difference between groups at the same timepoint. ^*^- as compared to baseline; # - compared to 4 weeks.

The clinical efficacy of the treatment in both groups of patients is presented in Figure 2. The results of the study showed that in both groups of patients with UC, the treatment was effective in most patients. Clinical response in the form of a significant decrease in stool frequency and a tendency to normalize its consistency after 4 weeks was detected in 14 (51.9%) patients in the standard care group and 16 patients (61.5%) of the FMT group (*p* = 0.583). However, in 5 (18.9%) patients of the standard care group, to achieve this intermediate effect, a slight escalation of treatment was required (increasing the dose of mesalazine to 4 g/day), which was significantly higher as compared to FMT, which were only 1 (3.5%) patient required escalation (*p* = 0.049). After 8 weeks, the main primary endpoint was achieved in 70.4 % of patients in the standard care group as compared to 84.6% of participants who received FMT as add-on therapy (*p* = 0.215). After 4 weeks, the Mayo score in the standard care group was 3.59 ± 1.21, and in the FMT group−3.15 ± 1.04 (*p* = 0.166) (Figures 2A, C). After 8 weeks of therapy, we observed a more pronounced decrease in Mayo score in the FMT group as compared to the standard care group (1.34 ± 1.44 vs. 2.14 ± 1.4; *p* = 0.045) (Figures 2A, C). The same findings for the current endpoint were confirmed in between group ANCOVA analysis (Table 2). All patients also showed a significant decrease in the level of fecal calprotectin (Figures 2B, D) compared to baseline, which correlated with clinical data, stool frequency, and clinical remission. At the same time, even in patients who reached clinical remission after 8 weeks, the level of fecal calprotectin remained elevated (72.15 + 10.45 in the standard care group and 70.92 + 10.68 μg/g in the FMT group). In between group analysis, fecal calprotectin changed insignificantly (*p* = 0.575, Table 2).

**Table 2:** Outcomes compared within and between groups.

	**Standard care group (*n* = 27)**	**FMT group (*n* = 26)**
**Mayo score**		
Baseline value	5.03 ± 0.80	5.00 ± 0.80
Week 8 value	2.14 ± 1.40	1.34 ± 1.44
*p*-value for change from	<0.001	<0.001
baseline		
Between-group *p*-value	0.048	
**Calprotectin feces**		
Baseline value	265.12 ± 47.63	256.36 ± 47.68
Week 8 value	72.15 ± 10.45	70.92 ± 10.68
*p*-value for change from	<0.001	<0.001
baseline		
Between-group *p*-value	0.575	

For within-group analysis paired sample t-tests were used. ANCOVA was used to identify any differences between the two groups after intervention, adjusting for baseline value.

### Secondary outcomes analysis

We also analyzed the effect of basic therapy and FMT on the gut microbiota composition in patients with UC in terms of secondary outcomes analysis (Table 3). Changes in the qualitative and quantitative composition of the gut microbiota were recorded in most patients with UC before the start of treatment In patients with left-sided UC with moderate disease activity, there was a decrease in the number of *Bacteroidetes* and *Firmicutes* with the growth of *Actinobacteria* and other opportunistic bacteria namely *Proteobacteria*. Accordingly, the Firmicutes/Bacteroidetes (F/B) ratio was 0.64. Four weeks after the start of treatment, a change in the ratio of the main microbial phenotypes was recorded. In all patients, an increase in the number of *Bacteroidetes* and *Firmicutes* was noted. The level of *Bacteroidetes* in the FMT group returned to normal, and the abundance of *Firmicutes* almost reached the normal value and was significantly higher as compared to baseline only in the FMT group (31.5 vs. 23.0%, *p* < 0.05). Normal value was obtained from analysis of microbiota composition in Ukrainian population, fecal concentrations of *Bacteroidetes, Firmicutes, Actinobacteria* and *Firmicutes/Bacteroidetes* (F/B) ratio were analyzed in 61 adult individuals (28). It should be noted that the increase in the *Bacteroidetes* and *Firmicutes* and the decrease in *Actinobacteria* and other representatives of opportunistic bacterias in patients after FMT were significantly higher as compared to the standard care group. In addition, after FMT we observed a significant increase in the abundance of butyrate-producing *F. prausnitzii*, which may also indicate an improvement in gut microbiota composition (Table 3).

**Table 3:** Contents of the main phylotypes of microorganisms in patients with UC at baseline and 4 weeks after treatment (%).

**Microbial phylotype (%)**	**Standard care group (*****n*** = **27)**		**FMT group (*****n*** = **26)**	

	**Baseline**	**After 1 month**	**Baseline**	**After 1 month**
*Bacteroidetes*	35.0	38.0	36.0	42.1
*Firmicutes*	24.0	26.1	23.0	31.5^*^
*Actinobacteria*	23.0	25.9	24.0	19.2^*^
Other	18.0	10.0	17.0	7.2^*^
*F/B* Ratio	0.68	0.68	0.64	0.75
*Faecalibacterium prausnitzii*	3.0	3.2	3.1	4.3^*^

^*^ p < 0.05 as compared to baseline values.

Thus, the clinical efficacy of treatment in both groups of patients was accompanied by an improvement in gut microbiota composition, which was significantly more pronounced in the group of patients with UC who additionally underwent FMT. We believe that the microbiological efficacy of FMT in patients with mild/moderate UC is associated with a modification of the metabolic activity of the gut microbiome due to the high content of the donor of regulatory molecules and metabolites in the feces, which led to a significant increase in the level *Firmicutes* and *Bacteroidetes* and thereby increasing the synthesis of short-chain fatty acids, in particular butyrate.

### Adverse events

Adverse events (AEs) likely related to FMT were stated in patients with UC. No serious AEs were noted. In the FMT group, 6 patients experienced AE. Most often, there was a short-term increase in abdominal pain and bloating (3 patients), 2 patients has complaints of diarrhea, and 1 of constipation. In the standard care group, 1 patient exhibited constipation, and another one had headaches. All AEs reported by patients were estimated as mild in their intensity and disappeared spontaneously. The overall incidence of AEs was higher for FMT but was comparable between groups (23.1 vs. 7.4%, *p* = 0.113).

## Discussion

Thus, according to the results obtained, a single FMT improved the results of basic UC therapy with mesalazine, which manifested itself in the form of an insignificant larger number of patients with the clinical response after 4 weeks, which was associated with significantly less amount of patients who required treatment escalation. The clinical remission rate was more pronounced in the FMT group and characterized by a greater decrease in the Mayo score after 8 weeks as compared to the standard care group. Unfortunately, fecal calprotectin, despite its pronounced decrease, did not completely normalize within the treatment periods in both groups, which indicates the need for prolongation of basic therapy.

Our data are consistent with the results of several controlled studies indicating the effectiveness of FMT in patients with active UC. Thus, Moayyedi et al. blindly randomized 70 patients with active UC who received either allogeneic FMT in enemas or water enemas (control) (20). Primary endpoints, such as a decrease in total Mayo score of <3 and endoscopic healing (0 on the endoscopic Mayo scale) after 6 weeks were recorded in 24% of patients who received FMT and 5% of patients who received placebo. Interestingly, the majority of patients who had an effect received FMT from one donor (39 vs. 10% from other donors), which confirms the critical role of donor selection (20). Paramsothy et al. studied the effectiveness of FMT performed by colonoscopy, in patients with mild/moderate UC, while most patients received FMT by introducing material from several donors (from 3 to 7) (22). Steroid-free remission and endoscopic response or remission were achieved in 11 of 41 (27%) patients treated with active fecal material and 3 of 40 (8%) patients treated with placebo (saline). The clinical response was associated with an increase in CM diversity, and the lack of effect was associated with a relative increase in *Fusobacterium*. Costello et al.studied the effectiveness of FMT in patients with mild/moderate UC by repeated administration of frozen fecal material from several donors in enemas (21). At the same time, results were obtained compared with the previous study (remission in 32% of patients treated with fecal material vs. 9% in patients treated with placebo). The LOTUS study, the first which used oral FMT as maintenance therapy in UC, assessed donor engraftment's long-term effectiveness with clinical, endoscopic, and histological outcomes (29). The primary outcome was corticosteroid-free clinical remission with endoscopic remission or response at week 8. At week 8, FMT responders were randomly assigned to either continue or withdraw FMT for a further 48 weeks. At week 8, 53% of patients in the FMT group achieved the primary endpoint as compared to 15% in the placebo group (*p* = 0.027; OR 5.0, 95% CI 1.8–14.1) (29). All patients who continued FMT in the open-label phase were in clinical, endoscopic, and histologic remission at week 56 compared with none of the patients who had FMT withdrawn (29).

A systematic meta-analysis was conducted to assess FMT as a treatment for active UC in 277 participants. FMT was connected with better remission between four RCTs than placebo (30). A most recent meta-analysis involving 6 RCT and 324 patients with UC demonstrated that compared with placebo, FMT has a significant benefit in inducing combined clinical and endoscopic remission (OR 4.11; 95% CI 2.19–7.72; *p* < 0.0001). Subgroup analyses of influencing factors showed no differences between fresh or frozen FMT (*p* = 0.35) and different routes or frequencies of delivery (*p* = 0.80 and *p* = 0.48, respectively) (31). In contrast, a recent meta-analysis, with the inclusion of 14 RCT found that fresh (40.9%) as compared to frozen (32.2%) FMT can increase clinical remission rates in IBD patients, with no significant risk of study heterogeneity (I^2^ = 38%, *p* = 0.03) (32).

In our study, the clinical efficacy of treatment in both groups of patients was accompanied by an improvement in gut microbiota composition, which was significantly more pronounced in the group of patients with UC who additionally underwent FMT. It should be noted that the *Firmicutes* phylotype includes one of the main representatives of the obligate *Lactobacillus*, which plays a significant role in the formation of colonization resistance and stability of the gut microbiome. In addition, representatives of *Firmicutes* have a significant effect on the metabolic activity of the gut microbiota, taking part in the synthesis of short-chain fatty acids, including butyrate, thereby modifying the state of the intestinal mucosal barrier (33, 34). The number of *F. prausnitzii* belonging to the family Ruminococcaceae, a member of the *Firmicutes* phylotype, is considered a regulatory and plays an important role in maintaining intestinal homeostasis, was also significantly reduced before treatment (*p* < 0.05). It's believed that decreased *Firmicutes* and *Faecalibacterium prausnitzii* in patients with UC can be an unfavorable prognostic sign and a marker of the severity of changes in the gut microbiome (11). The number of *Actinobacteria* in patients with UC was significantly higher than in healthy individuals, this is because the *Actinobacteria* family includes many representatives of opportunistic microbiota, the number of which increases with intestinal dysbiosis associated with UC.

So, FMT is an emerging treatment strategy for UC. Clinical research on FMT in treating gastroenterological diseases has dramatically increased in the last few years and is still ongoing. However, there are many issues to solve before FMT can become standard therapy for UC, including donor selection, administration routes, frequencies, easy-to-administer formulation development, and optimal patient population (35).

## Conclusion

Even single transplantation of fecal microbiota (fresh material) bears the potential to be a well-tolerated and safe method of treatment in a large number of patients with mild-to-moderate UC, contributing to an increase in the effectiveness of basic therapy after 4 and 8 weeks, as well as a significant improvement in the abundance of the gut microbiota as early as 4 weeks after FMT. The addition of FMT to the standard therapeutic protocols for UC warrants efficacy at reaching clinical improvement and preservation of gut eubiosis, in line with the goals of precision medicine.

In our opinion, the effectiveness of FMT depends primarily on the microbial composition and quality of the donor material used (from one or several donors; fresh or frozen material), the number of procedures (single or repeated FMT), routes of administration of the material (colonoscopy, enemas, naso-duodenal probe), previous treatment, the prevalence of the process and severity of UC. Therefore, future studies are recommended to further characterize these parameters and develop the necessary guidelines to routinely add FMT to the treatment options for UC.

## Data availability statement

The raw data supporting the conclusions of this article will be made available by the authors, without undue reservation.

## Ethics statement

The studies involving human participants were reviewed and approved by Ethics Committee at Ukrainian Research and Practical Center of Endocrine Surgery, Transplantation of Endocrine Organs and Tissues of the Ministry of Health of Ukraine (protocol number: 6/2020). The patients/participants provided their written informed consent to participate in this study.

## Author contributions

ST, AD, and NK contributed to the conceptualization and the original idea of this manuscript. ST, AD, OT, IK, and NK contributed to methodology and reviewed the literature. ST, AD, LA, OK, and TF involved in validation and revised and validated the literature findings. IK and OT performed formal analysis. ST and AD contributed to investigation. ST and NK involved in data curation, did writing–original draft preparation, and did writing–review and editing. ST, OT, and IK did visualization. ST, LB, LA, and NK involved in supervision. ST and IK contributed to project administration. All authors contributed to the article and approved the submitted version.
